# Integrated transcriptome analysis and combinatorial machine learning to construct a homeostatic model of acetylation for ccRCC and validate the key gene GCNT4

**DOI:** 10.1186/s12935-025-03837-4

**Published:** 2025-06-25

**Authors:** Baohua Zhu, Ziyang Mo, Yi Bao, Xinxin Gan, Linhui Wang

**Affiliations:** 1 Department of Urology, The First Affiliated Hospital, Naval Medical University, Second Military Medical University, Shanghai, China; 2 Department of Urology, The Third Affiliated Hospital, Naval Medical University, Second Military Medical University, Shanghai, China

**Keywords:** Clear cell renal cell carcinoma, Acetylation, Prognosis, Machine learning, GCNT4

## Abstract

**Background:**

Clear cell renal cell carcinoma (ccRCC) is one of the most common malignant tumors of the urinary system. Protein acetylation plays a key role in regulating cellular processes and cancer signaling pathways. This study explores the potential biological mechanisms of ccRCC from the perspective of acetylation.

**Methods:**

This study obtained RNA-seq data and clinical information of ccRCC from TCGA and ICGC, and single-cell RNA sequencing datasets from the GEO database. Ten machine learning algorithms and their 101 combinations were used to analyze the prognostic significance of acetylation-related differentially expressed genes (DEGs) and to construct a prognostic risk model. GSEA was used to analyze the enrichment of different signaling pathways in high-risk and low-risk groups, and the correlation between immune infiltration and risk scores was assessed. Finally, the function of the key gene GCNT4 was verified through cell experiments.

**Results:**

This study identified 84 acetylation-regulated key genes with significant expression differences between tumor and normal tissues, closely linked to patient prognosis. The LASSO + RSF combination model performed best, and the model could accurately predict patient prognosis. The survival of patients in the high-risk group was significantly worse than that in the low-risk group. High expression of GCNT4 was associated with better survival prognosis and was expressed at higher levels in normal tissues than tumor tissues. Overexpression of GCNT4 significantly inhibited the proliferation, invasion, and migration of renal cancer cells and may affect acetylation by regulating the levels of O-GlcNAc modification in cells.

**Conclusion:**

This study constructed a ccRCC acetylation homeostasis model via transcriptome analysis and machine learning, validating GCNT4 as a key gene. High expression of GCNT4 is associated with better survival prognosis and affects acetylation by regulating O-GlcNAc modification levels, inhibiting the proliferation and migration of renal cancer cells, providing a new potential target for the treatment of ccRCC.

**Supplementary Information:**

The online version contains supplementary material available at 10.1186/s12935-025-03837-4.

## Introduction

Clear cell renal cell carcinoma (ccRCC) is one of the three most common malignant tumors of the urinary system [1], and it is the most prevalent histological subtype of renal cancer, accounting for 85% of renal cancer cases [2]. Additionally, the global incidence rate is still on the rise [3]. Although ccRCC is a disease that can be detected early and successfully treated through surgery, up to one-third of cases will develop metastases, leading to fatal outcomes [4]. The first-line treatment for ccRCC includes immunotherapy using programmed cell death 1 (PD-1) checkpoint inhibitors, such as nivolumab and pembrolizumab, combined with molecular targeted therapy. The most commonly used molecular targeted drugs for RCC patients are tyrosine kinase inhibitors that block vascular endothelial growth factor receptors (VEGFR), such as axitinib, cabozantinib, and lenvatinib [5–8]. However, the options for targeted therapy in ccRCC are still very limited, and there is an urgent need for in-depth research into the biological mechanisms of ccRCC.

Protein acetylation is an important post-translational modification that involves the transfer of an acetyl group from acetyl coenzyme A (Ac-CoA) to specific lysine residues on proteins [9]. This modification plays a critical role in regulating various cellular processes, including gene transcription, DNA repair, and metabolic pathways [10]. In the context of cancer, protein acetylation has become a key factor because it is involved in the regulation of oncogenic and tumor-suppressive signaling pathways. Abnormal acetylation levels can lead to the dysregulation of these pathways, thereby promoting the occurrence, progression, and metastasis of tumors [11]. For example, acetylation can modulate the activity of transcription factors and metabolic enzymes, thereby affecting tumor metabolism and the tumor microenvironment. Additionally, the dynamic balance between histone acetyltransferases (HATs) and histone deacetylases (HDACs) is crucial for maintaining cellular homeostasis, and its disruption can promote oncogenesis. Therefore, targeting protein acetylation pathways has become a promising strategy for cancer treatment, with HDAC inhibitors showing potential in clinical applications [12]. Acetyl-CoA within the cell is closely related to the acetylation process [13], and changes in Acetyl-CoA levels directly affect histone acetylation, thereby regulating transcription, genomic integrity, and replication. At the same time, histone acetylation also directly replenishes the metabolite pool for redistribution in chromatin acetylation or other metabolic reactions [14]. Both non-enzymatic and enzymatic acetylation of Acetyl-CoA play important roles in protein acetylation [15].

Protein acetylation plays a significant role in the occurrence and development of ccRCC, and histone acetylation modifications are closely related to the development of ccRCC [16]. In ccRCC, abnormal levels of histone acetylation can lead to the dysregulation of gene expression, thereby promoting the proliferation and metastasis of tumor cells [17]. Furthermore, deacetylases such as SIRT3 are downregulated in ccRCC, and their low expression is associated with tumor progression. SIRT3 regulates mitochondrial function and metabolic pathways through deacetylation modifications, and its overexpression can enhance mitochondrial biogenesis and inhibit the growth of ccRCC [18]. Emerging studies reveal critical roles of protein acetylation in ccRCC suppression: SFMBT2 stabilizes via self-acetylation, inhibiting tumor growth [19]. ACLY acetylation enhances enzymatic activity, fueling acetyl-CoA overproduction and lipid accumulation in ccRCC [20]. Hypoxia-induced ERRα acetylation synergizes with HIF signaling to drive malignant ccRCC transformation [21]. These findings illuminate the interplay between epigenetic modifications and microenvironmental cues in tumor progression, highlighting novel therapeutic targets for ccRCC.

Acetylation plays a significant role in the life activities of tumors, but there is still a lack of systematic research on it in ccRCC. Existing studies have focused on the acetylation of individual proteins or specific energy substances such as lipid metabolism. This study investigated acetylation-related gene features at multiple omics levels. We used single-cell and bulk transcriptomes to identify relevant genes and then employed a new machine learning framework that combines 10 machine learning algorithms and their 101 combinations to construct relevant features. We selected GCNT4 as the key gene in the model and demonstrated the tight association of the model with the prognosis and immune status of ccRCC. Subsequently, we conducted gene intervention of GCNT4 at the cellular level to verify its effects on acetylation and ccRCC proliferation.

## Materials and methods

### Data collection and processing

RNA-seq data for ccRCC and corresponding clinical information were obtained from The Cancer Genome Atlas (TCGA) with the International Cancer Genome Consortium (ICGC). Transcripts were extracted for transcripts per kilobase million (TPM) values for subsequent analysis, and genes with average expression below 0.1 were excluded. In addition, we retrieved ccRCC single-cell RNA sequencing datasets GSE210038, GSE222703, and GSE224630 from the Gene Expression Omnibus (GEO) database. Specifically, GSE210038 contains single-cell RNA sequencing data from the tumors of seven patients with ccRCC and two samples of normal adjacent tissue. GSE222703 contains single-cell RNA sequencing data for three ccRCC patient tumors and fresh samples of adjacent healthy tissues. GSE224630 contains scRNA-seq data for tumor samples from five untreated ccRCC patients.

Single-cell RNA sequencing data were processed using the Seurat package (v4.0.2) in R. Gene expression matrices were generated via Read10X and converted to a Seurat object using CreateSeuratObject. Quality control thresholds included: (1) retention of genes detected in ≥ 3 cells; (2) inclusion of cells expressing 300-7,000 genes; (3) exclusion of cells with ≥ 5% mitochondrial gene content. Data were normalized and subjected to PCA. Cell clusters were identified using FindNeighbors and FindClusters (resolution = 1.2, empirically determined) based on the top 30 principal components. UMAP projections (RunUMAP) visualized cluster relationships. Cell types were annotated via canonical markers: tumor cells (CA9, NNMT, PAX8); epithelial cells (GATM, ALDOB); mast cells (MS4A2); B cells (MZB1, CD79A, MS4A1); macrophages (CD14, CD68); monocytes (C1QA, CD68); NK/NKT cells (PTPRC, NKG7, KLRD1); mesangial cells (PDGFRB); and endothelial cells (CD34).To analyze differentially expressed genes (DEGs), the “FindMarkers” function in the Seurat software package was used. Statistical significance of DEGs was determined using the Wilcoxon test (*p* < 0.05), and other parameters were set to default values. Acetylation scores for each cell type were assessed using the UCell tool in the irGSEA software package. Univariate Cox regression analysis was used to identify acetylation-associated genes in the TCGA and ICGC cohorts. Kidney tissue immunohistochemistry (IHC) images obtained from Human Protein Atlas (HPA).

### Construction and validation of prognostic signature based on integrated machine learning approaches

The TCGA and ICGC dataset was used as a training cohort for screening prognosis-related genes and developing prognostic features (Table 1). Ten machine learning algorithms and their 101 different combinations for variable selection and model construction based on a ten-fold cross-validation framework were analyzed to determine the prognostic significance of acetylation-related degs in the TCGA and ICGC cohort. Ten machine learning algorithms including Lasso, random survival forest (RSF) stepwise Cox, partial least squares regression for Cox (plsRcox), Ridge, elastic net (Enet), CoxBoost, generalized boosted regression modeling (GBM), survival support vector machine (survival-SVM), and supervised principal components (SuperPC).

**Table 1:** Baseline clinical features the TCGA dataset

	Level	Overall
n		539
Stage_S (%)	Stage_I	271 (50.56)
	Stage_II	59 (11.01)
	Stage_III	123 (22.95)
	Stage_IV	83 (15.49)
Stage_T (%)	T1	277 (51.39)
	T2	71 (13.17)
	T3	180 (33.40)
	T4	11 (2.04)
Stage_N (%)	N0	242 (44.90)
	N1	16 (2.97)
	NX	281 (52.13)
Stage_M (%)	M0	430 (80.07)
	M1	79 (14.71)
	MX	28 (5.21)
Race (%)	asian	8 (1.48)
	Black or african american	54 (10.02)
	Not reported	7 (1.30)
	white	470 (87.20)
Gender (%)	Female	186 (34.51)
	Male	353 (65.49)
Age (mean (SD))		60.666 (12.114)

The prognostic model used overall survival (OS), disease-specific survival (DSS) and progression-free survival (PFS) as survival endpoints. Using lasso and RSF regression models, the most informative prognostic markers among the candidate DEGs were identified to construct the prognostic risk model. And they were categorized into high-risk and low-risk groups based on risk scores. Univariate Cox regression analysis was performed using the R packages survminer and survivor. Survival analysis was performed using the Kaplan-Meier (KM) method and statistical significance was assessed using the log-rank test. The time-related area under the receiver operating characteristic curve was calculated using the “timeROC” software package. Risk scores, survival status and the area under the curve (AUC) of receiver operating characteristic (ROC) over time were analyzed, to assess the predictive performance of the model in predicting survival outcomes in kidney cancer patients.

### Gene set enrichment analysis (GSEA)

Quantify the enrichment scores of specific gene sets in the samples using GSEA. The enrichment of different signaling pathways in the high and low risk groups was obtained. Signaling pathways with significant differences were then analyzed using the “limma” package. GO and Hallmark pathway enrichment analysis based on GSEA and Hallmark gene set data.

### Immune infiltration analysis

Immune infiltration results were analyzed and correlations with risk scores were assessed using the R software package immunedeconv. The CIBESORT with ESTIMATE algorithm was used to quantify the relationship between 22 immune cell infiltrations and risk scores and to predict the level of immune cell infiltration in tumor tissue.

### Cell culture

Human renal cancer cell lines: OS-RC-2 cell line, A-498 cell line and 786-O cell line (human clear cell renal carcinoma cell line) were obtained from Wuhan Pricella Biotechnology Co., Ltd. All cell lines were identified using short tandem repeat assay. Cells were cultured in DMEM complete medium at 37 °C with 5% CO2. GCNT4 overexpression cell lines groups were treated with OSMI-1 (HY-119738, MedChemExpress) (50 µ M) for 24 h.

### CCK8 experiment

Cell viability was detected by CCK8 assay. OSRC2 cells, 786-O cells and A498 cells were inoculated in 96-well plates at a concentration of 4 × 10^3^ cells/well and incubated for 0–96 h, then 10µL CCK8 reagent was added. After incubation for 3 h, the absorbance (OD value) at 450 nm was measured by MicroplateReader.

### Construction and transfection of lentivirus

GCNT4 overexpressing (OE) and negative control (Ctrl) lentiviruses were obtained from Shanghai Genechem Co.,Ltd. At 48 h after infection, the lentiviruses were treated with 2 µg/ml puromycin (Beyotime, China) for 3–7 d. Stable transfectants were obtained, and then the expression of GCNT4 was analyzed by western blotting.

### Western blot

Total cellular proteins were obtained using a total protein extraction kit (ProteinTech). Proteins were separated by sodium dodecyl sulfate-polyacrylamide gel electrophoresis and transferred to NC membranes. After being closed with 5% skim milk powder for 1 h at room temperature, the membranes were incubated overnight at 4 °C with the appropriate antibodies, including: GCNT4 (1:1000, Signalway Antibody #45802), O-GlcNAc (1:1000, Abcam ab2739), GAPDH (ProteinTech). After incubation of NC membranes with appropriate secondary antibodies, the membranes were visualized using a Tanon 5200 system and ECL detection reagents and quantified using ImageJ software.

### Tanswell migration and invasion assays

100 µL of Matrigel (diluted 1:8 in serum-free medium) was spread evenly into the upper chamber of the Transwell and allowed to solidify (not required for migration experiments). Cells (4 × 10^4^ cells/well) from the serum-free medium mix were inoculated evenly into the upper Transwell. 600 µL of DMEM medium was added to the lower Transwell. After 48 h of incubation, the cells were fixed in 4% paraformaldehyde and stained with 0.2% crystal violet. The unstained cells were wiped away. Acquiring images with a microscope and count the infiltrated cells in the lower chamber.

### Wound healing experiments

Firstly, three horizontal lines were drawn evenly on the back of the 6-well culture plate with a marker along the straightedge as a marking line. About 5 × 10^5^ logarithmic growth phase cells were added to each well. After 24 h of incubation, a vertical scratch (perpendicular to the scratch line on the back side) was made on the cells with the pipette tip, and the cells were rinsed with PBS to remove the scratched cells. Then the medium was added and incubated. Photographs of the cells at the same location were taken at 0 h and 24 h, respectively. The wound healing ability of the cells was analyzed using Image J software.

### Determination of Acetyl-CoA content

Cells were assayed for acetyl coenzyme A (Acetyl-CoA) content using the Acetyl-CoA Content Assay Kit(Beijing Solarbio Science & Technology Co., Ltd.).

### Statistical analysis

Statistical analysis and visualization were performed using the R program (version 4.0.1). GraphPad Prism 9.0 software (GraphPad software, Inc.) and SPSS software were used for statistical analysis. Each experiment was repeated three times and the results were expressed as mean ± standard deviation (SD). p-value < 0.05 was considered statistically significant. Statistical significance was as follows: *P < 0.05, ** *P* < 0.01, *** *P* < 0.001.

## Results

### Identification of acetylated DEGs

**Fig. 1:**
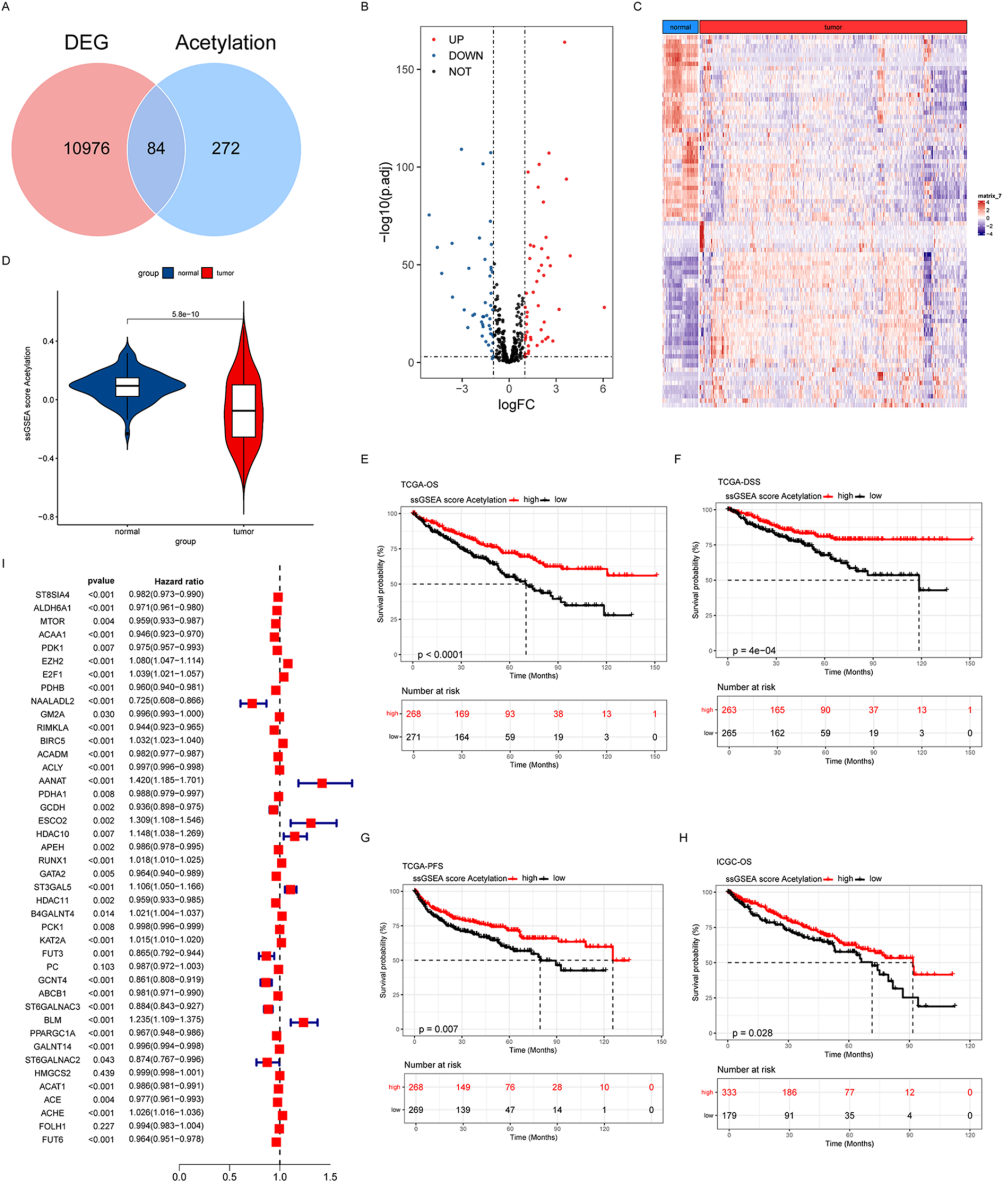
Identification of acetylated DEGs. (**A**) Venn diagram described the acetylated gene set intersecting with the DEGs (tumor tissues and corresponding paracancer normal tissues in the TCGA database). (**B**) Volcano diagram of gene expression levels of the intersecting genes, including up-regulated (red), down-regulated (blue) and genes with no significant changes (black). (**C**) Heatmap of the expression levels of the intersecting genes in normal tissues and renal cancer tissues. (**D**) Violin plots of acetylation scores in normal versus renal cancer tissues based on ssGSEA. (**E**) Relationship between acetylation scores and patients’ overall survival (OS) in the TCGA dataset. (**F**) Relationship between acetylation score and patient disease-specific survival (DSS) in the TCGA dataset. (**G**) Relationship between acetylation score and patient progression-free survival (PFS) in the TCGA dataset. (**H**) Relationship between acetylation score and patient OS in the ICGC dataset. (**I**) Forest plot of one-way Cox regression analysis for screening acetylation genes associated with prognosis

We initially extracted the differentially expressed genes (DEGs) of tumor tissues and corresponding paracancerous normal tissues from the TCGA database and subsequently performed an intersection analysis with the set of acetylated genes. This procedure aimed to identify the key genes that may be regulated by acetylation during tumorigenesis and development. The results indicated that 84 genes were present in both DEGs and acetylated gene sets, suggesting that these genes may play significant roles in the acetylation regulatory network of tumors (Fig. 1A). Utilizing volcano plots and heat maps, we demonstrated the expression changes and patterns of intersecting genes. The volcano plot illust.rates the relationship between the log2 fold change (logFC) of differentially expressed genes and the adjusted p-value (-log10(p.adj)) of the negative logarithmic transformation, emphasizing the genes with significant expression changes. Heat maps, conversely, visualized the expression levels of these intersecting genes across different samples, thereby elucidating their expression heterogeneity (Fig. 1B and C). Using single sample Gene Set Enrichment Analysis (ssGSEA), we quantified the intersecting genes to evaluate the overall activity of the acetylated gene set in the samples. Violin plots illustrating the distribution of acetylation scores in tumor tissues compared to paracancerous tissues demonstrate disparities in acetylation activity between tumor and normal tissues (Fig. 1D). We further analyzed the relationship between acetylation scores and patients’ overall survival (OS), disease-specific survival (DSS), and progression-free survival (PFS) in the TCGA dataset. The Kaplan-Meier survival curves demonstrated a statistically significant difference in the probability of survival between patients with high scores and those with low scores, indicating that acetylation activity may be strongly associated with the prognosis of tumor patients (Fig. 1E-H). Similarly, we conducted an analysis of the relationship between the acetylation score and overall patient survival in the ICGC dataset. The results demonstrated a significant association between the acetyl score and the survival probability of patients, further substantiating the role of acetylation in tumor prognosis. Finally, we performed one-way Cox regression analysis to screen for acetylation genes associated with prognosis. The results showed that multiple acetylation-related genes were significantly associated with patients’ prognosis (*p* < 0.05), and these genes may serve as potential prognostic markers or therapeutic targets (Fig. 1I).

### Development and validation of prognostic features based on integrated machine learning approaches

**Fig. 2:**
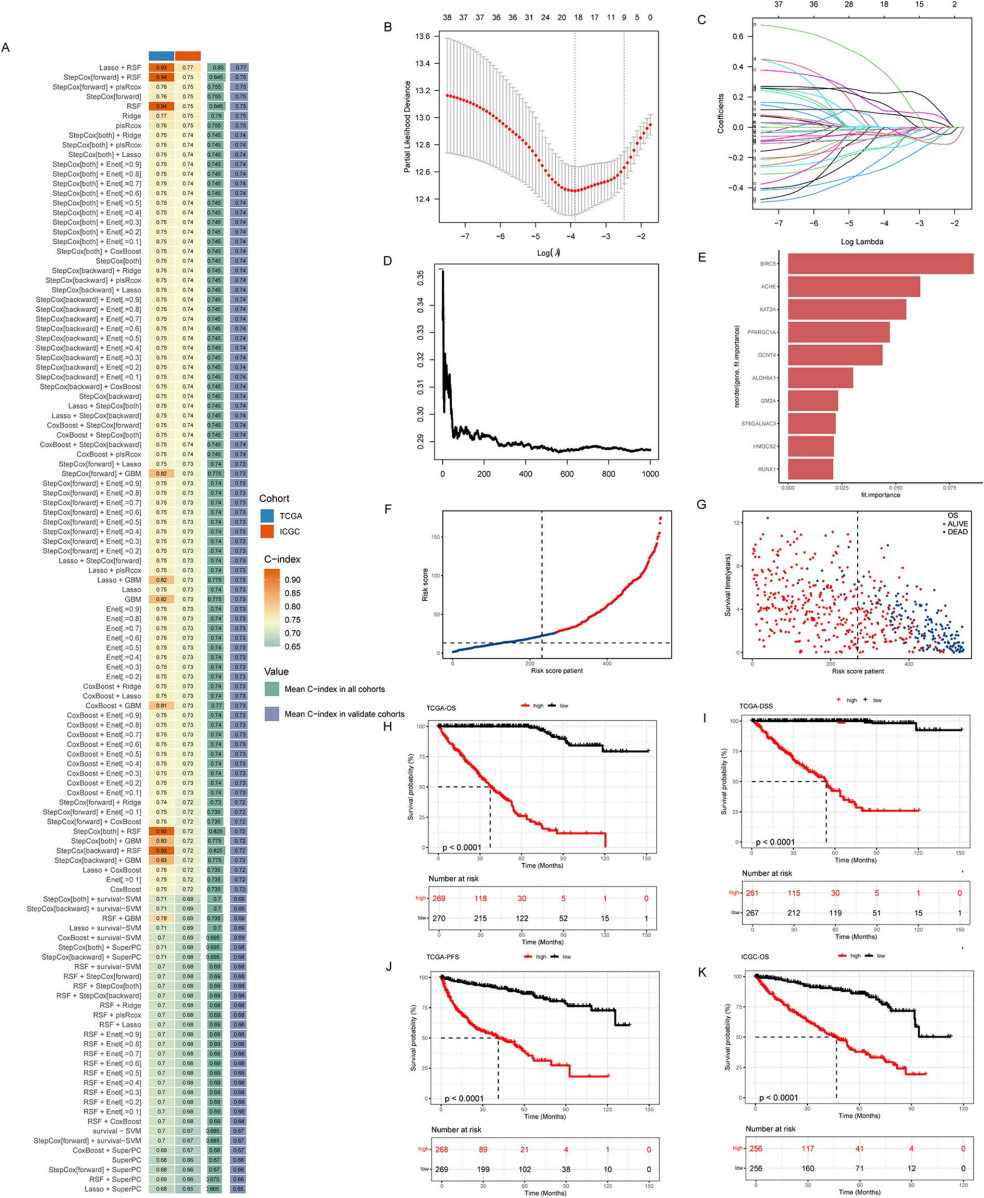
Development and validation of prognostic features based on integrated machine learning methods. (**A**) Obtaining 101 kinds of prediction models by 10 machine learning methods and calculating the C-index. (**B**, **C**) Visualization of LASSO regression analysis in the TCGA cohort. (**D**, **E**) Visualization of RSF regression analysis in the TCGA cohort. (**F**) Risk score distribution of patients in the TCGA cohort. (**G**) Overall survival status of patients in the TCGA cohort. (**H**, **I**,** J**) Kaplan-Meier curves of OS, DSS and PFS for low-risk and high-risk groups in the TCGA dataset, based on the log-rank test. (**K**) Kaplan-Meier curves of OS for low-risk and high-risk groups in the ICGC dataset. The blue line represents low-risk patients and the red line represents high-risk patients

We applied 101 distinct combined machine learning methodologies to analyze the data in TCGA and ICGC datasets to determine which method exhibited the highest consistency index (C-index). The results of the analysis indicated that the LASSO + RSF combination demonstrated superior performance among all models evaluated, displaying the highest C-index value. Consequently, we selected the LASSO + RSF combination as the foundation for subsequent modeling (Fig. 2A). By applying LASSO regression analysis to the TCGA dataset, we identified genes associated with tumor prognosis. Figure 2B and C show the results of the LASSO analysis, where the coefficient plots show the relative importance of each gene in the prognostic model. These results help us understand which genes may have an important impact on the prognosis of patients with tumors. Random Survival Forest (RSF) analysis was also applied to TCGA dataset to assess the impact of different genes on patient prognosis. Figure 2D and E show the results of RSF analysis, revealing the complex relationship between genes and survival time, and identifying genes that have a significant impact on prognosis. Based on the results of the LASSO + RSF analysis, we constructed a prognostic risk model and categorized patients into high-risk and low-risk groups based on risk scores. Figure 2F and G demonstrate that the survival of patients in the high-risk group was significantly worse than that of patients in the low-risk group, indicating that our risk model could accurately predict the prognosis of patients. We conducted further analysis of OS, DSS and PFS for patients categorized into high- and low-risk groups within the TCGA dataset. The Kaplan-Meier survival curves presented in Fig. 2H-J demonstrated that patients in the high-risk group exhibited significantly lower survival probabilities compared to those in the low-risk group across all three survival metrics, thereby providing additional validation for the efficacy of our risk modeling approach. Finally, we performed an OS analysis in the ICGC dataset for patients in the high- and low-risk groups. The survival curves in Fig. 2K similarly showed that patients in the high-risk group had a poorer survival prognosis, which is consistent with the results in the TCGA dataset, suggesting that our risk model has a better ability to generalize across different datasets.

### Evaluation of the homeostatic model of acetylation

**Fig. 3:**
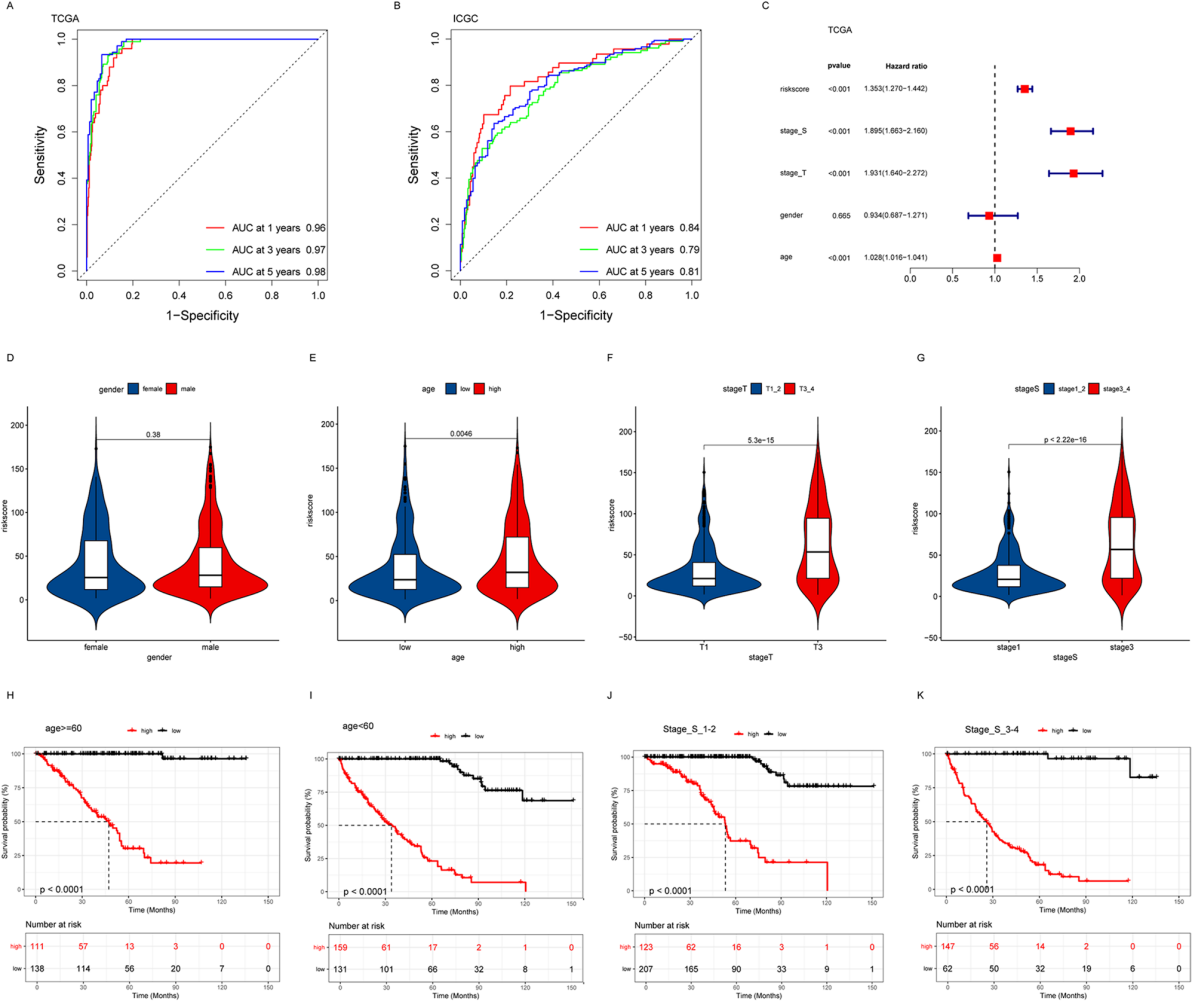
Evaluation of the risk model. (**A**, **B**) ROC curves showing the specificity and sensitivity of the risk model for predicting OS at 1, 3, and 5 years in the TCGA training set and the ICGC dataset. (**C**) Forest plot of the distribution of clinical characteristics according to the risk scores. (**D**-**G**) Differences in risk scores between patients grouped by gender, age, stage S and stage T. (**H**-**K**) Kaplan-Meier curves of OS for ccRCC patients aged ≧ 60 years, aged < 60 years, and with tumor stages S1-2 and S3-4

The area under the receiver operating characteristic curve (ROC) was utilized to assess the predictive power of the risk model. Figure 3A illustrates the AUC values of the risk model in the TCGA dataset of 0.96, 0.97, and 0.98 at 1, 3, and 5 years, respectively, demonstrating exceptionally high predictive accuracy. Figure 3B, conversely, exhibits the corresponding AUC values in the ICGC dataset as 0.84, 0.79, and 0.81, respectively, which are marginally lower than those in the TCGA dataset, yet still indicate robust predictive performance. These findings suggest that the risk model can effectively differentiate the survival prognosis of diverse patients. We further analyzed the correlation between patients’ clinical information, including gender, age, and tumor stage, and risk scores. The relationship between these clinical characteristics and risk scores is shown in the forest plot (Fig. 3C) and bar charts (Figs. 3D-G), which show the distribution of risk scores for patients of different sexes, age groups, and tumor stages. Notably, we observed that older patients (Fig. 3E) and patients with advanced tumors (Fig. 3G) exhibited higher risk scores, which may be associated with a less favorable survival prognosis, whereas the effect of gender on survival risk was not significant. Finally, we divided the patients into different subgroups, including patients aged greater than 60 and less than 60 years, as well as tumor stages S1-2 and S3-4, and performed overall survival (OS) analyses. The Kaplan-Meier survival curves in Figs. 3H-K showed that patients with older (Fig. 3H and I) and advanced tumors (Fig. 3J and K) had a significantly worse survival prognosis, consistent with their higher risk scores. These results further confirm the clinical relevance of our risk model and suggest that age and tumor stage are important factors that influence patient prognosis.

### Underlying molecular mechanisms of the acetylation-related signature in bulk transcriptome

**Fig. 4:**
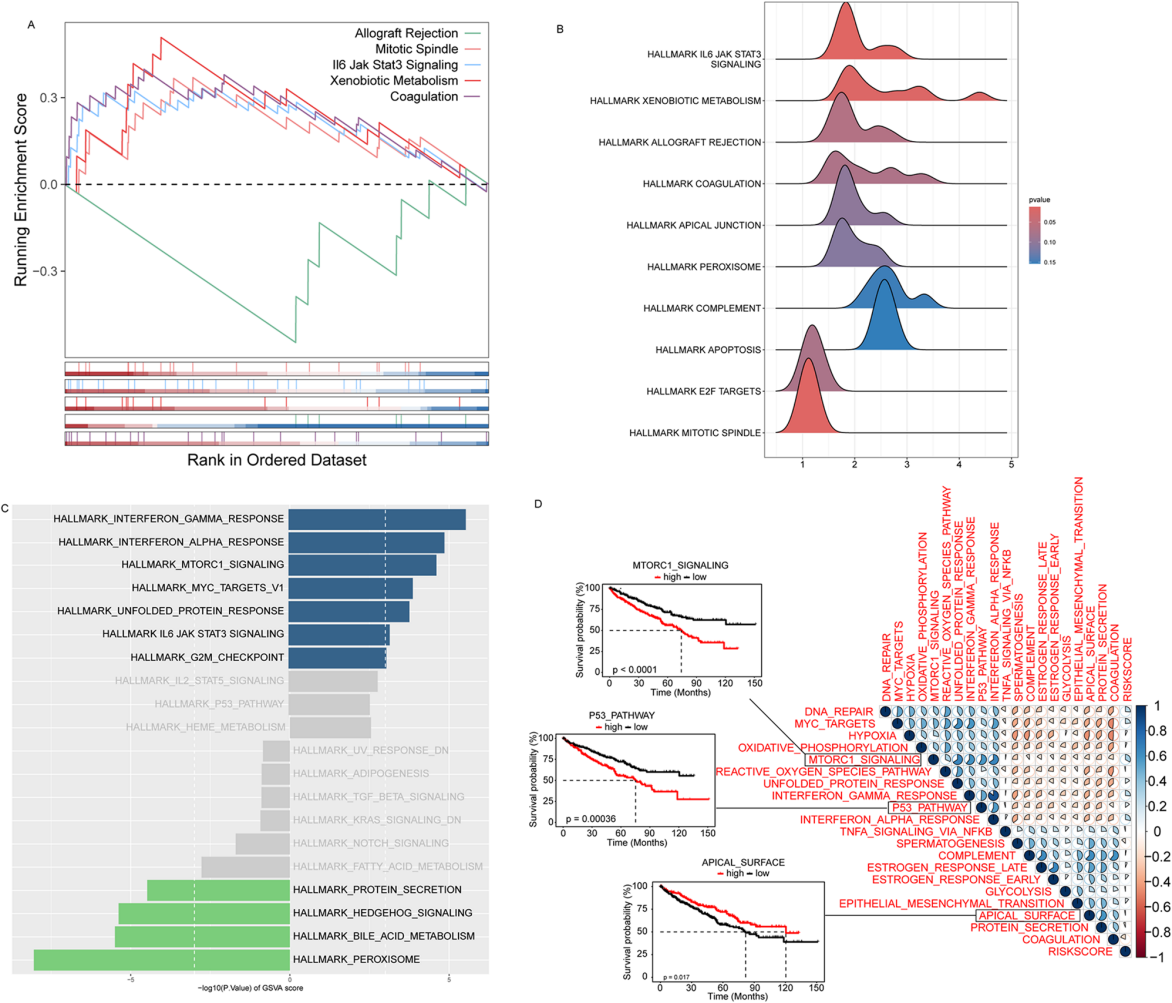
Characterization of the acetylation-related transcriptome in ccRCC patients. (**A**, **B**) GO terms enriched in the differential genes between the high-risk group and low-risk group by GSEA analysis. (**C**) Differences in hallmark pathway activities between the high-risk and low-risk groups scored by GSVA. (**D**) Correlation between the OS of patients and the activity of the pathway by GSVA. Significant correlations between the OS and GSVA scores of APICAL surface, P53 pathway, and MTORC1 signaling wereshown by Kaplan-Meier survival curves

Next, the molecular mechanisms underlying the correlation between the acetylation-related signature and ccRCC prognosis were further investigated. We first divided the tumor samples into high-risk and low-risk groups based on their risk scores. We then extracted the differential genes between the two groups and used GSEA analysis based on the GO gene set to explore the biological processes and signaling pathways involved in these differential genes. Figure 4A and B demonstrate the results of GSEA analysis, where the enrichment score (NES) and P-value indicate the degree of enrichment of different signaling pathways in the high-risk and low-risk groups. The results showed that multiple HALLMARK signaling pathways were significantly different between the high- and low-risk groups, suggesting that these pathways may play important roles in tumor development and prognosis. Such as defense responses to bacterium and other organisms, humoral immune responses, immunoglobulin Complex, adaptive immune response, etc.

Furthermore, we scored the differential genes in each sample using the Gene Set Variation Analysis (GSVA) method to quantify the activity of each sample on specific signaling pathways. Figure 4C illustrates the results of GSVA scoring, and statistical difference analysis was performed to determine which signaling pathways were significantly different between the high- and low-risk groups. These results provide important information for understanding the molecular heterogeneity of tumors and may reveal new therapeutic targets. Figure 4D illustrates the top 20 HALLMARK signaling pathways exhibiting the highest GSVA scores and analyzes the correlation between the activity of these pathways and the overall survival (OS) of patients. The Kaplan-Meier survival curves demonstrated that elevated activity of specific signaling pathways (e.g., APICAL surface, P53 pathway, and MTORC1 signaling) is associated with a less favorable survival prognosis for patients, whereas increased activity of other pathways may be associated with an improved prognosis. These findings underscore the potential significance of specific signaling pathways in tumor prognosis and may inform future therapeutic strategies.

### Association of the prognostic risk model with immune cell infiltration

**Fig. 5:**
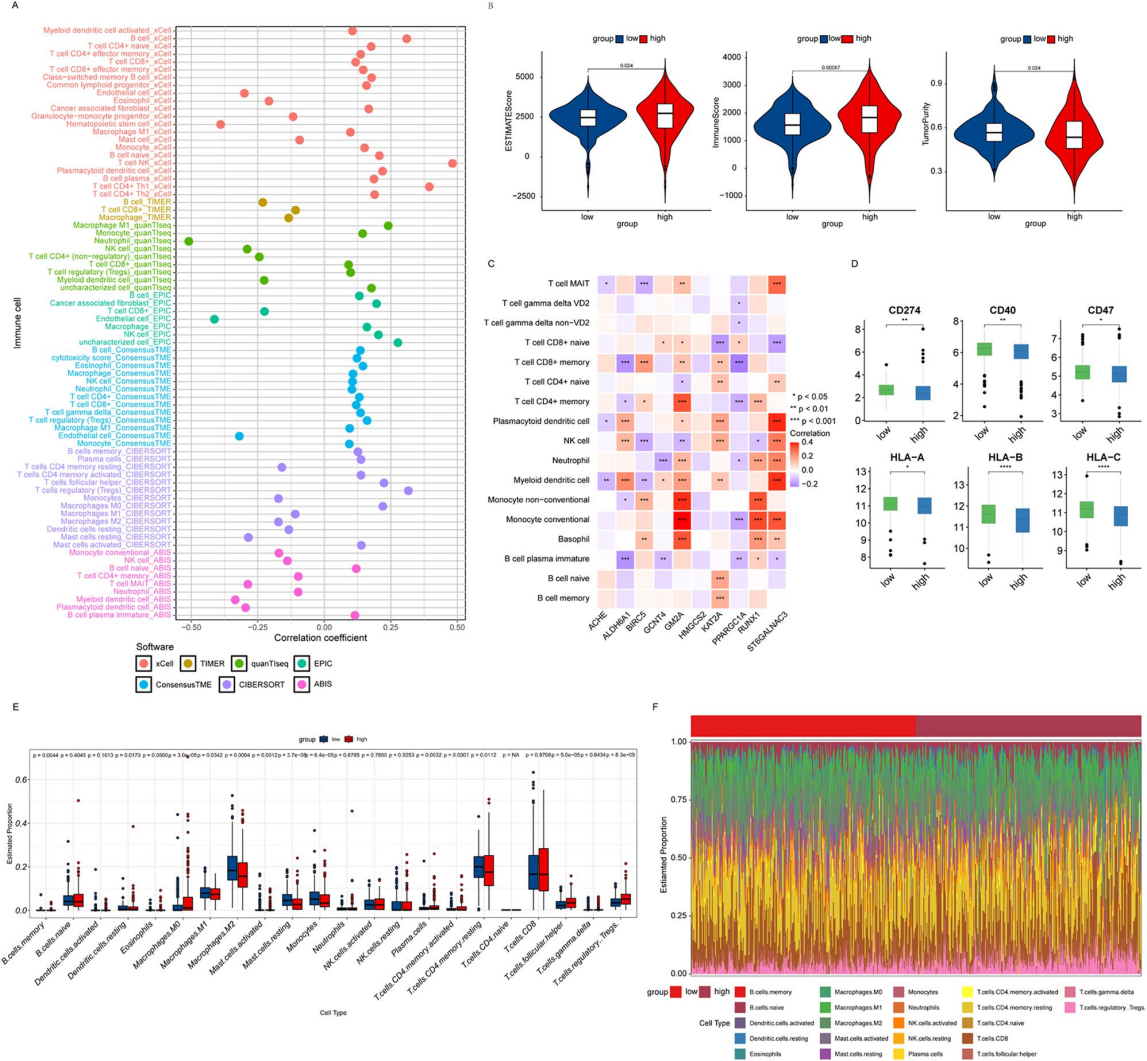
Immune Infiltration Analysis. (**A**) Correlations between 7 immune cell infiltration outcomes and risk scores were analyzed using the immunedeconv software package. (**B**) Violin plots of the differences in ESTIMATE score, immune score, and tumor purity in the high-risk and low-risk groups. (**C**) Correlation analysis between model genes and immune cell infiltration levels. (**D**) Differences in expression of key immune checkpoint genes in the high-risk and low-risk groups. (**E**, **F**) Box plots and histograms showing the distribution and expression differences of the immune cell subpopulations in the high-risk group versus the low-risk group by the CIBESORT algorithm

Immune cell infiltration plays a crucial role in the development of ccRCC. Subsequently, we further utilized a variety of algorithms and software tools to assess the relationship between immune cell infiltration and the prognostic risk model, and explored the distribution characteristics of immune cell infiltration in different risk groups. Figure 5A demonstrates the correlation coefficients and p-values between different immune cell subpopulations and risk scores. The results showed a significant correlation between multiple immune cell types (e.g., T cells CD4+, NK cells, B cells, T cells regulatory (Tregs), etc.) and risk scores. Thus revealing a potential link between immune cell infiltration and tumor prognosis. We assessed the level of immune cell infiltration in the tumor samples using the ESTIMATE algorithm and classified the patients into high-risk and low-risk groups based on the risk score. Figure 5B shows the differences in immune cell infiltration levels between the two groups. The results showed that the high-risk group displayed higher infiltration levels in certain immune cell subpopulations, which may be associated with immune escape from the tumor and prognosis.

Further, we explored the correlation between model genes and the level of immune cell infiltration. Figure 5C demonstrates the correlation coefficients and statistical significance between these genes (ACHE, ALDH6A1, BIRC5, GCNT4, GM2A, HMGCS2, KAT2A, PPARGC1A, RUNX1 and ST6GALNAC3) and immune cell subpopulations. These results help to understand how specific genes affect immune cell infiltration and function. Furthermore, we analyzed the expression levels of immune checkpoint genes in the high- and low-risk groups. Figure 5D showed the differences in the expression of key immune checkpoint genes (e.g., CD274, CD40, CD47, HLA-A, HLA-B, and HLA-C) between the two groups. The expression levels of these genes may influence the immune response in the tumor microenvironment and thus correlate with patient prognosis.

Finally, we analyzed the level of immune cell distribution between the high-risk and low-risk groups using the CIBERSORT algorithm. Figure 5E and F demonstrate the distribution of different immune cell subpopulations and expression differences in the two groups. These results further confirmed the heterogeneity of immune cell infiltration in the tumor microenvironment and may reveal immune cell subpopulations associated with tumor progression and prognosis.

In Supplementary Fig. 2, our analysis uncovered significant correlations between GCNT4 expression and pivotal immune microenvironmental characteristics in TCGA. Specifically, GCNT4 demonstrated negative correlation with Tumor Immune Dysfunction (TIDE) and Exclusion scores (*P* = 0.02). Additionally, GCNT4 was inversely associated with myeloid-derived suppressor cell (MDSC) infiltration (*P* = 4.16 × 10 − 7) and immune exclusion signatures (*P* = 1.59 × 10^− 4^). Conversely, positive correlations were noted with CD274 (*P* = 3.59 × 10^− 9^) and Interferon Gamma (IFNG) (*P* = 5.9 × 10^− 3^).

### Molecular mechanism of GCNT4 involved in renal carcinogenesis

**Fig. 6:**
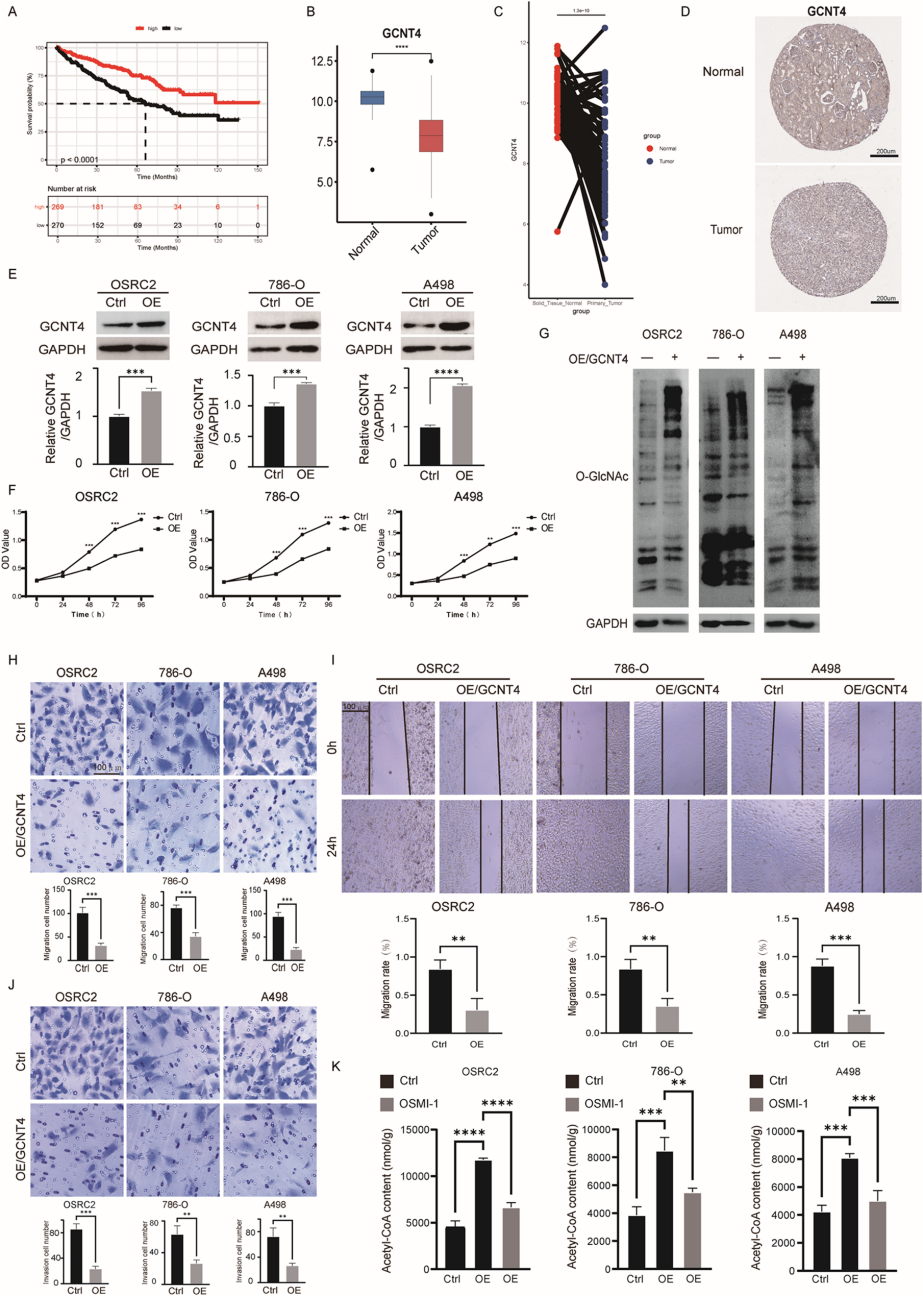
Molecular mechanisms of GCNT4 involvement in renal carcinogenesis. (**A**) Kaplan-Meier survival curves showing the correlation between GCNT4 and overall survival of patients. (**B**, **C**) Box plot and scatter plot showing the differences in GCNT4 expression in normal and tumor tissues. (**D**) IHC images showing GCNT4 expression in normal and tumor tissues. (**E**) GCNT4 overexpression in OSRC2, 786-O and A498 cells was detected using western blot (*n* = 3, compared with Ctrl group, Unpaired t-test). (**F**) OD values of OSRC2, 786-O and A498 cells cultured for 0–96 h in control and overexpression groups, for assessment of cell proliferation. (*n* = 3, compared with Ctrl group, Unpaired t-test) (**G**) Western blot detection of O-GlcNAc expression in control versus overexpression group. (**H**) Transwell assay to detect the number of migrating cells (magnification, ×100). (*n* = 3, compared with Ctrl group, Unpaired t-test) (**I**) Wound healing assay to detect wound healing ability (magnification, ×100). (*n* = 3, compared with Ctrl group, Unpaired t-test) (**J**) Transwell assay to detect the number of cell invasion (magnification, ×100). (*n* = 3, compared with Ctrl group, Unpaired t-test) (**K**) Acetyl-CoA content assay. (*n* = 3, compared with Ctrl group, Unpaired t-test)

Among the homeostatic model genes for acetylation, we selected GCNT4 as the key differential gene and targeted it for genetic intervention to validate its effectiveness. The Kaplan-Meier survival curves (Fig. 6A) showed that the survival probability of the GCNT4 high-expression group (red) was significantly higher than that of the low-expression group (black), suggesting that high GCNT4 expression may be associated with a better survival prognosis. We then examined the differences in GCNT4 expression in normal and tumor tissues. The Box plot (Fig. 6B) and scatter plot (Fig. 6C) of GCNT4 expression levels showed that GCNT4 expression levels were generally higher in normal tissues than in tumor tissues. As shown in the IHC images, the expression of GCNT4 was significantly lower in tumor tissues compared to normal tissues (Fig. 6D). These results suggested that the reduced expression of GCNT4 may be associated with tumor development.

After that, we explored the role of GCNT4 gene in renal carcinogenesis by overexpressing GCNT4 in three renal cancer cell lines (OSRC2, 786-O and A498 cell lines). We successfully constructed GCNT4 overexpression cell lines and detected the overexpression efficiency using western blotting (Fig. 6E). The results of CCK8 proliferation assay showed that the absorbance of all overexpression groups was significantly reduced and the cell viability was significantly reduced compared with the control group, revealing that GCNT4 could inhibit the proliferation of tumor cells (Fig. 6F). The results of transwell assay showed that the GCNT4 overexpression groups all had significant inhibition of tumor cell migration and invasion (Fig. 6H and J). The results of wound healing assay showed that the GCNT4 overexpression group had poor healing ability on OSRC2, 786-O and A498 cells. GCNT4 overexpression significantly inhibited wound healing (Fig. 6I). These results indicated that GCNT4 could inhibit the growth, invasion and migration of renal cancer cells.

Previous studies have shown that the main function of the GCNT family is to regulate protein glycosylation, which is closely related to intracellular O-linked β-N-acetylglucosamine (O-GlcNAc) modification. Therefore, we explored the molecular mechanism of GCNT4 in the development of renal cancer by detecting the level of intracellular O-GlcNAc modification. Western blot results showed that the overall expression level of intracellular O-GlcNAc modification in the overexpression group increased (Fig. 6G). Acetyl-CoA, an important metabolic intermediate, is involved in a variety of biosynthetic pathways. We measured the Acetyl-CoA content in the control groups, GCNT4 overexpression groups and GCNT4 overexpression treated with OSMI‑1 groups. The overexpression group of each cell line was found to show significantly higher Acetyl-CoA content than the control group, and also higher Acetyl-CoA content than GCNT4 overexpression groups treated with OSMI‑1 (Fig. 6K). This suggests that GCNT4 may regulate Acetyl-CoA levels by altering the level of O-GlcNAc modification of intracellular proteins.

### The correlation of the prognostic risk model with single‑cell characteristics

**Fig. 7:**
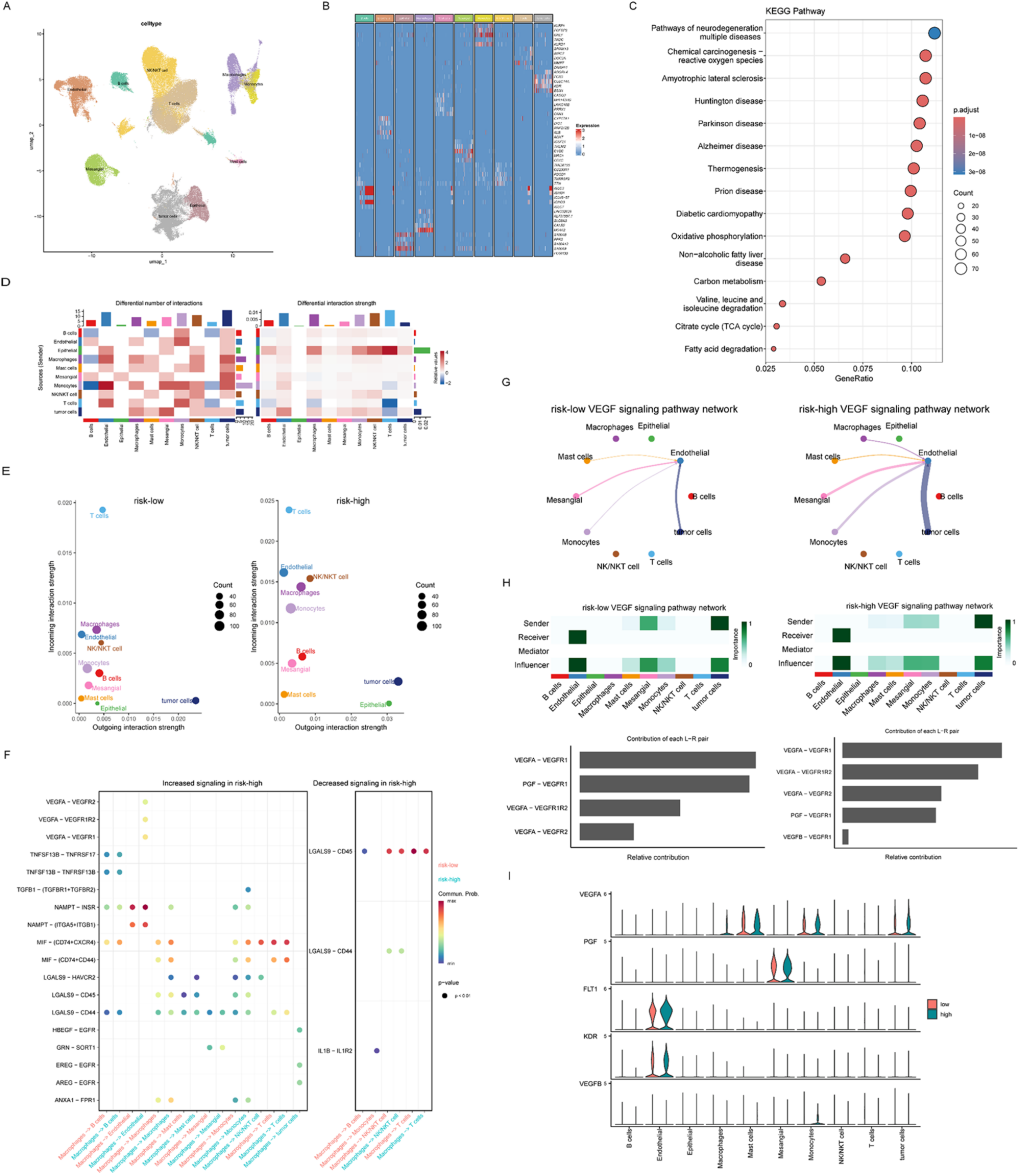
The correlation of the prognostic risk model with single‑cell characteristics. (**A**) t-SNE plot showing the cell type identification. (**B**) Heatmap showing specific gene expression profiles in different cell types. (**C**) KEGG pathway enrichment analysis. (**D**) Heatmap showing differences in the number and strength of interactions between different cell types. (**E**) Scatter plot showing incoming interaction strength and outgoing interaction strength between different cell types under low and high risk conditions. (**F**) Changes in signaling under high-risk conditions. (**G**, **H**) Vascular endothelial growth factor (VEGF) signaling pathway network. (**I**) Violin plot showing the distribution of expression of five molecules (VEGFA, PGF, FLT1, KDR, and VEGFB) in different cell types, versus the difference in expression between low and high risk conditions

In this study, we performed a combined analysis of single-cell RNA sequencing data from GSE210038, GSE222703, and GSE224630, to gain insights into the roles of different cell types in specific biological processes and their transcriptional regulatory mechanisms. We first performed a cluster analysis of the combined single-cell transcriptome data to identify different cell types. The findings demonstrated a clear distinction among various cell types, including NK/NKT cells, tumor cells, endothelial cells, mesangial, epithelial cells, monocytes, macrophages, T cells, mast cells, and B cells. The identification of these cell types establishes a foundation for further biological analyses (Fig. 7A). Also, We have supplemented the gene-celltype information in Supplementary Fig. 1. Furthermore, heatmaps were generated to visualize the expression patterns of the top five marker genes exhibiting the highest expression levels in each cell cluster. These heatmaps elucidated the specific gene expression characteristics of different cell types. For instance, marker genes for B cells may include immunoglobulin genes, whereas marker genes for endothelial cells may encompass angiogenesis and inflammatory response-related genes. These data provide molecular-level insights that contribute to the understanding of the functions of various cell types and their roles in disease processes (Fig. 7B).

We performed KEGG pathway enrichment analysis and cell signaling analysis. Figure 7C showed the gene expression data based on KEGG pathway analysis. The results showed a significant enrichment of genes related to metabolic pathways such as fatty acid degradation, citrate cycle (TCA cycle), Valine, leucine and isoleucine degradation, and oxidative phosphorylation. The heatmap demonstrated the differences in the number of interactions and the strength of interactions between different cell types (Fig. 7D). The scatter plots in Fig. 7E showed the incoming interaction strength and outgoing interaction strength between different cell types in the risk-low and risk-high conditions, respectively. The results showed that the cell-to-cell interaction strength increased significantly in the risk-high state.

Figure 7F demonstrated the changes in signaling under high-risk condition, which is divided into two parts: increased signaling and decreased signaling. We found that the signaling of VEGFA-VEGFR1, VEGFA-VEGFR2, MIF-(CD74 + CXCR4) and MIF-(CD74 + CD44) was significantly increased in the high-risk state; whereas the signaling of LGALS9-CD45, LGALS9-CD44, and IL1B-IL1R2 was decreased, which may affect the tumor cell adhesion and migration. Figure 7G and H demonstrated the VEGF signaling pathway network at low and high risk. We observed an altered pattern of cell-cell interactions and significant differences in the regulatory mechanisms of the VEGF signaling pathway at different risk levels. This may be related to disease progression or different pathological states, where cells may need to respond to more severe inflammatory and immune challenges by enhancing the activity of the VEGF signaling pathway under high-risk conditions. In addition, the VEGFA-VEGF1R2 combination plays an important role in signaling, with a higher contribution in the high-risk group, and a reduced contribution of PGF-VEGFR1 (Fig. 7H).The Violin plot in Fig. 7J showed the distribution of the expression of five molecules (VEGFA, PGF, FLT1, KDR and VEGFB) in different cell types and compared the difference in expression between low and high risk states. In the high-risk state, the expression levels of VEGFA were significantly increased in Macrophages, Mast cells, Monocytes and tumor cells; FLT1 and KDR were significantly increased in Endothelial; and PGF was significantly increased in Mesangial. Differences in the expression of these molecules may be associated with functional changes in cells, activation of signaling pathways, or biological markers of disease states.

Collectively, these data suggested that metabolic disorders and alterations in intercellular signaling may play a key role in disease progression. These findings provide new perspectives for understanding the metabolic and immunoregulatory mechanisms involved in disease progression and may provide potential targets for the development of new therapeutic strategies.

## Discussion

Clear cell renal cell carcinoma (ccRCC) is an increasingly serious global disease burden, and prognostic factors are crucial for estimating disease progression, selecting appropriate treatment methods, and determining overall survival rates. This study utilized a novel computational framework and collected expression files across multiple cohorts worldwide to explore the correlation between acetylation-related genes and ccRCC prognosis. Our model identified GCNT4 gene, termed acetylation differentially expressed genes, which play a significant role in prognosis.

Acetylation is highly dependent on acetyl-CoA [22], and this biological process is regulated by various enzymes [23]. Abnormal levels or activities of these rate-limiting enzymes can drive malignant transformation [24], including significant impacts on the tumor microenvironment [25]. In previous studies, the focus was often limited to the effects of one or a few key proteins on acetylation and acetyl-CoA, such as ACLY [26, 27] and the ACSS family [27–29]. In ccRCC, research focusing on acetylation is still limited, mainly to smaller aspects such as lipid metabolism [30].

This study is the first to integrate all acetylation-related genes and systematically analyze the significant role of intracellular acetylation-related genes in ccRCC prognosis at multiple omics levels using a fused machine model. This approach allowed for the identification of key genes that may have been overlooked. Among the acetylation differentially expressed genes, we selected GCNT4 as a key differential gene and conducted gene intervention to verify its effectiveness.

GCNT4 is a member of the β-1,6-N-acetylglucosaminyltransferase (GCNT) family, which primarily regulates protein glycosylation. Research in the oncology field is abundant, such as the correlation between GCNT1 expression and disease aggressiveness in prostate cancer [31]; GCNT2 as a biomarker and novel therapeutic target for melanoma [32], and its induction of epithelial-mesenchymal transition in colorectal and esophageal cancer [33, 34]; GCNT3 is mainly associated with the prognosis of colon cancer but also relates to pancreatic cancer and hepatocellular carcinoma [35]; GCNT4 is closely related to gastric cancer [36]. Glycosylation is the most common, complex, and dynamic post-translational modification of lipids and proteins, essential for every biological process [37]. We noted that the GCNT family possesses important acetylglucosaminyltransferase activity [38], closely related to intracellular glucosamine (O-GlcNAc) modification. O-GlcNAc modification has been shown to contribute to various cellular functions, including signal transduction, protein localization and stability, transcription, chromatin remodeling, mitochondrial function, and cell survival [39]. Dysregulation of O-GlcNAc is the basis of multiple metabolic disorders leading to human diseases, including cancer, neurodegenerative diseases, and diabetes [40], with its underlying mechanisms being highly complex. For example, almost all enzymes in glycolysis have been shown to undergo O-GlcNAc modification, with the O-GlcNAc modification of glucose-6-phosphate dehydrogenase regulating pentose phosphate pathway activity, and the O-GlcNAc modification of GSK3β regulating glycogen synthesis [41]. In adipocytes, activation of the HBP and increased O-GlcNAc levels also stimulate fatty acid oxidation [42]. Its regulatory function in various metabolisms suggests potential roles in clear cell renal cell carcinoma. This study demonstrated that after overexpression of GCNT4, the proliferation, invasion, and migration of three renal cancer cell lines (OSRC2, 786-O, and A498) were significantly inhibited. We hypothesized that this alteration was caused by changes in intracellular acetyl-CoA levels and tested that intracellular acetyl-CoA levels were significantly elevated in overexpressing GCNT4. Mechanistically, we measured intracellular O-GlcNAc modification levels and found that GCNT4 regulates acetyl-CoA levels by altering the O-GlcNAc modification levels of intracellular proteins, thereby affecting acetylation.

Our findings suggest a potential combinatorial strategy involving GCNT4 modulation to improve anti-PD-L1 therapeutic outcomes, proposing a distinct perspective for future investigation. GCNT4-high tumors exhibit a “hotter” immunological profile, evidenced by negative associations with TIDE and immunosuppressive myeloid-derived suppressor cells (MDSCs). Mechanistically, reduced immune exclusion suggests GCNT4 facilitates T-cell infiltration through extracellular matrix remodeling (e.g., hyaluronan degradation). Paradoxically, while GCNT4 drives PD-L1 upregulation via IFN-γ signaling—a pathway typically linked to adaptive resistance—this occurs alongside robust baseline T-cell activity, creating a unique vulnerability. PD-L1 blockade in this context may amplify pre-existing antitumor immunity, akin to the enhanced efficacy observed in IFN-γ-rich tumors like MSI-H colorectal cancer [43].

The crosstalk among reactive oxygen species (ROS), metabolic pathways, and acetylation has been documented across diverse species, including cyanobacteria, plants, and mammals. In our single-cell dataset, KEGG pathway enrichment analysis revealed significant alterations in metabolic pathways such as oxidative phosphorylation, the TCA cycle, and lipid metabolism, alongside ROS-related signaling pathways—a finding consistent with prior research. VEGF, a pivotal mediator of tumor angiogenesis, exhibits functional interplay with acetylation signaling. For instance, H3 histone acetylation regulates VEGF transcription, while VEGF itself stimulates angiogenesis via ETS1 acetylation. A clinical study in colorectal cancer demonstrated that combining histone deacetylase inhibitors with anti-VEGF monoclonal antibodies enhances therapeutic efficacy. Our findings further highlight adaptive activation of VEGF signaling in high-risk clear cell renal cell carcinoma (ccRCC), potentially driving angiogenic or vascular remodeling responses to inflammatory and immune stressors. These observations suggest that dual targeting of acetylation pathways and VEGF signaling may represent a promising therapeutic strategy for ccRCC, warranting further mechanistic exploration and clinical validation.

However, several limitations of this study need to be considered. First, the detailed mechanism by which GCNT4 regulates intracellular O-GlcNAc modification levels remains to be elucidated. Our study only demonstrated that overexpression of GCNT4 leads to an increase in intracellular O-GlcNAc modification levels. Importantly, while our findings suggest that GCNT4 may regulate Acetyl-CoA levels through modulating protein O-GlcNAcylation, the causal relationship and molecular intermediates connecting these two metabolic nodes remain obscure. Whether GCNT4 directly acts as a functional enzyme for protein O-GlcNAc modification or influences it through other signaling pathways is still unclear. Crucially, it is unknown whether the observed Acetyl-CoA alteration is a direct consequence of O-GlcNAc-mediated metabolic reprogramming, or occurs through parallel regulatory mechanisms. Future studies may incorporate multi-omics techniques, such as proteomics and metabolomics, combined with isotope tracing approaches to systematically investigate both the molecular mechanisms underlying GCNT4-mediated O-GlcNAc modification. Second, this study only conducted mechanistic research on GCNT4, which is less reported and less functionally clear among acetylation homeostasis differentially expressed genes. This is still not comprehensive for overall acetylation research in ccRCC and requires further exploration. Third, our results are only based on a limited number of cell lines. Additionally, the lack of in vivo experiments limits the translatability of functional findings. Moreover, the limited sample size of single-cell data may compromise the accuracy of cell subpopulation classification and DEG identification, potentially affecting the robustness and generalizability of prognostic models. Future studies should expand sample diversity, perform in vivo validation and increase single-cell data volume to enhance reliability and precision. Last, the study lacks clinical sample validation, particularly multi-center cohort studies or clinical tissue trials, limiting the prognostic model’s applicability in real clinical settings. Future studies should validate the model using clinical samples and multi-center trials to enhance its clinical utility and promote its translation into practice.

Despite these limitations, this study provides new insights into the molecular mechanisms of ccRCC occurrence and development from the perspectives of single-cell transcriptomics and bulk transcriptomics, especially by combining the prominent metabolic heterogeneity of ccRCC with acetylation, offering a novel integrated perspective. Through combined machine learning, we identified GCNT4 as a key gene regulating acetylation in ccRCC. Mechanistically, GCNT4 affects intracellular O-GlcNAc modification levels, thereby altering acetylation and influencing the outcome of ccRCC.

## Electronic supplementary material

Below is the link to the electronic supplementary material.

Supplementary Material 1: Supplementary fig. 1: The gene-celltype information. Cell types were annotated via canonical markers: tumor cells (CA9, NNMT, PAX8); epithelial cells (GATM, ALDOB); mast cells (MS4A2); B cells (MZB1, CD79A, MS4A1); macrophages (CD14, CD68); monocytes (C1QA, CD68); NK/NKT cells (PTPRC, NKG7, KLRD1); mesangial cells (PDGFRB); and endothelial cells (CD34).

Supplementary Material 2: Supplementary fig. 2: Correlations between GCNT4 expression and immune features in TCGA. (A) TIDE (negative, *P* = 0.02), (B) Exclusion (negative, *P* = 1.59 × 10^− 4^), (C) MDSC infiltration (negative, *P* = 4.16 × 10^− 7^), (D) CD274 (positive, *P* = 3.59 × 10^− 9^), and (E) IFNG (positive, *P* = 5.9 × 10^− 3^)

## Data Availability

The datasets generated during and/or analysed during the current study are available in the The Cancer Genome Atlas (TCGA), The International Cancer Genome Consortium (ICGC) and The Gene Expression Omnibus (GEO) database repository.
