# Supplementary figures and images for: Integrated transcriptome analysis and combinatorial machine learning to construct a homeostatic model of acetylation for ccRCC and validate the key gene GCNT4
Source: Cancer Cell Int. 2025 Jun 25;25:236. doi: 10.1186/s12935-025-03837-4 (PMC12199489; doi:10.1186/s12935-025-03837-4)

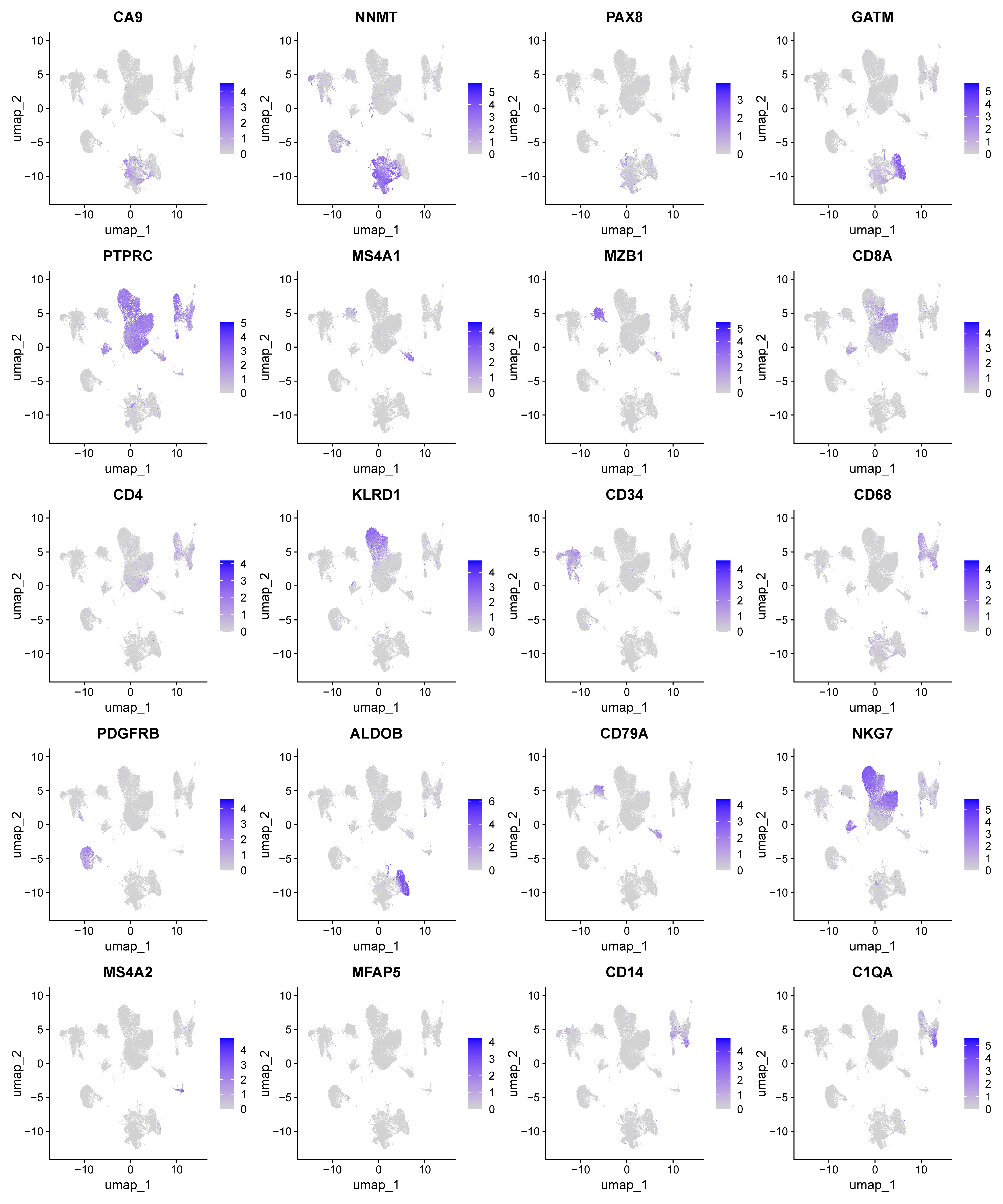

Supplement: Supplementary file 1 — Supplementary Material 1: Supplementary fig. 1: The gene-celltype information. Cell types were annotated via canonical markers: tumor cells (CA9, NNMT, PAX8); epithelial cells (GATM, ALDOB); mast cells (MS4A2); B cells (MZB1, CD79A, MS4A1); macrophages (CD14, CD68); monocytes (C1QA, CD68); NK/NKT cells (PTPRC, NKG7, KLRD1); mesangial cells (PDGFRB); and endothelial cells (CD34). [file 12935_2025_3837_MOESM1_ESM.tif]

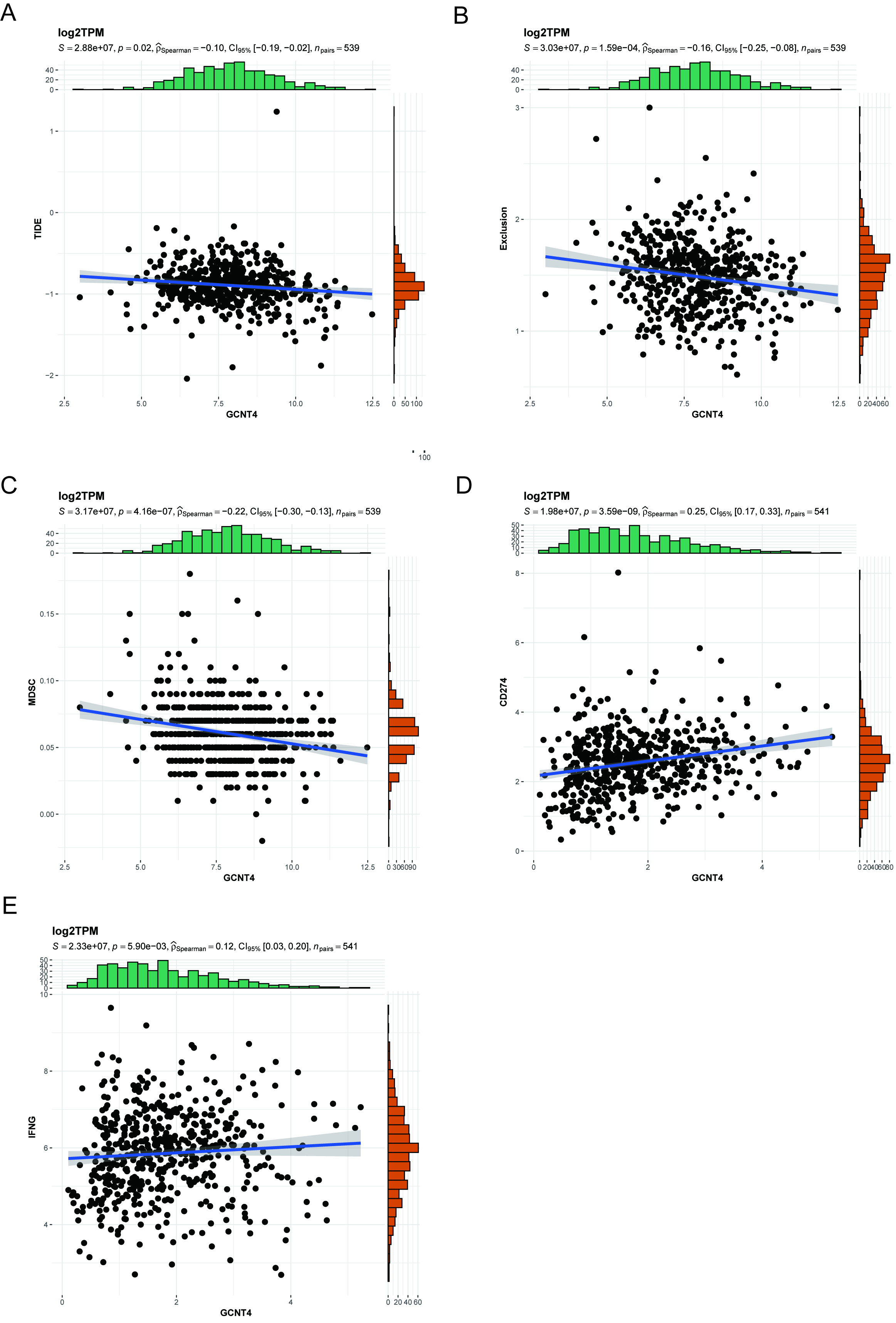

Supplement: Supplementary file 2 — Supplementary Material 2: Supplementary fig. 2: Correlations between GCNT4 expression and immune features in TCGA. (A) TIDE (negative, P = 0.02), (B) Exclusion (negative, P = 1.59 × 10− 4), (C) MDSC infiltration (negative, P = 4.16 × 10− 7), (D) CD274 (positive, P = 3.59 × 10− 9), and (E) IFNG (positive, P = 5.9 × 10− 3) [file 12935_2025_3837_MOESM2_ESM.tif]
